# Simulating the Spread of Peste des Petits Ruminants in Kazakhstan Using the North American Animal Disease Spread Model

**DOI:** 10.1155/2023/7052175

**Published:** 2023-03-28

**Authors:** Kairat Yessenbayev, Yersyn Mukhanbetkaliyev, Gulzhan Yessembekova, Ablaikhan Kadyrov, Akhmetzhan Sultanov, Aslan Bainiyazov, Temirlan Bakishev, Joseph Nkamwesiga, Fedor Korennoy, Sarsenbay Abdrakhmanov

**Affiliations:** ^1^S. Seifullin Kazakh Agrotechnical University, Nur-Sultan (Astana), Kazakhstan; ^2^Kazakh Research Veterinary Institute, Almaty, Kazakhstan; ^3^Dahlem Research School of Biomedical Sciences, Department of Veterinary Medicine, Freie Universität Berlin, Berlin, Germany; ^4^International Livestock Research Institute, Animal and Human Health Program, Kampala, Uganda; ^5^Federal Center for Animal Health (FGBI ARRIAH), Vladimir, Russia

## Abstract

In this study, we simulated the potential spread of Peste des Petits Ruminants (PPR) between small ruminant (SR) farms in the Republic of Kazakhstan (RK) in case of the disease's introduction into the country. The simulation was based on actual data on the location and population of SR farms in the RK using the North American Animal Disease Spread Model (NAADSM). The NAADSM employs the stochastic simulations of the between-farm disease spread predicated on the SIR compartmental epidemic model. The most important epidemiological indicators of PPR, demography of SR farms, and livestock management characteristics in the RK were used for model parameterization. This article considers several scenarios for the initial introduction of PPR into the territory of Kazakhstan, based on previously identified high-risk regions and varying sizes of initially infected farms. It is demonstrated that the duration and size of the outbreak do not depend on the size of initially infected farms but rather depend on the livestock concentration and number of farms in the affected area. This implies that the outbreak may affect the largest number of farms in the case of introduction of the disease into farms in southern Kazakhstan. However, even in the most unfavorable scenario, the total number of affected farms does not exceed 2.4% of all SR farms in the RK. The size of the affected area is, in most cases, no larger than an averaged 2-level administrative division's size, which suggests the scale of a local epidemic. The chosen model provides ample opportunity to study the impact of different control and prevention measures on the spread of PPR as well as to assess the potential economic damage.

## 1. Introduction

Peste des Petits Ruminants (PPR) is one of the most devastating and highly contagious animal diseases, epidemics of which cause widespread economic damage, reduce the investment attractiveness of the agro-industrial complex, and reduce a country's export potential. The economic damage to goat and sheep breeding is extremely high. The mortality rate in primary outbreaks can reach 100% and up to 50% in secondary outbreaks. Goats are the most susceptible to PPR, and their mortality rate can reach 95% [1, 2].

Direct economic damage is caused by animal deaths, production losses (milk yields, meat quality and weight gain, and wool and fur losses), and the cost of quarantine measures. Indirect economic losses result from international trade ban on such small ruminants since PPR is a notifiable transboundary animal disease. Animal deaths are mainly due to complications from secondary infections of the respiratory organs affected by PPR. The Food and Agriculture Organization of the United Nations (FAO) estimates the global annual economic damage caused by this disease at more than 2 billion US dollars [3].

With the world's population anticipated to reach 9.7 billion by 2050, the breeding of small ruminants is expected to increase to match the rising demand for animal-source food such as meat and milk. This will likely generate new opportunities for livestock producers and traders, thus increasing governments' and industry interest to invest in strengthening small ruminant value chains. Due to the significant socio-economic damage and negative impact on food security in many countries around the world, PPR is included in the list of priority diseases of the five-year plan of action of the FAO/WOAH World Framework Program for the progressive control of transboundary animal diseases [3].

In PPR endemic countries, the disease has negative impacts on sources of livelihood, food security, and economic activity of the entire population including livestock producers, consumers, and traders. As such, the global agenda for control and eradication of PPR by 2030 is poised to create a significant positive impact on the productivity of small ruminants and livelihoods of people [3, 4].

As of 2022, PPR has not been officially registered in Kazakhstan; however, given its widespread in the world, there is a real threat of PPR introduction into new areas including the territory of the RK [5–7]. The inadequacy of biosecurity measures among the countries neighboring the Republic of Kazakhstan makes it more than urgent to evaluate the potential of introduction and subsequent spread of PPR in the territory of Kazakhstan. The analysis of PPR epidemic situation and trends in the disease spread in recent years in neighboring countries indicates a considerable threat of PPR introduction in Kazakhstan. Epidemic situation in Mongolia and China, as well as in Georgia, Turkey, and Iran, is of particular importance [8–13].

The socio-economic and environmental conditions in Kazakhstan have changed dramatically over the last 30 years. Consequently, there has been an emergence of a number of small, mixed holding management systems replacing the large, specialized cattle, sheep, and goat breeding farms. New trade and economic relations of Kazakhstan with Afghanistan, India, Iran, China, Mongolia, Pakistan, Turkey, and many other neighboring and distant countries have also been established. These have since led to a significant increase in transboundary migration of people and animals to and from Kazakhstan. Given the peculiarities of livestock management systems in Kazakhstan and the need to combat this dangerous disease, it is important to study the epizootic process and analyze the potential spread of PPR virus to improve preventive measures and preparedness in the context of Kazakhstan [8, 14].

Taking into account the fact that PPR has never been registered in the territory of the RK, the most appropriate method to assess the risk of the disease spread in the territory of the country following its introduction is simulating the PPR epizootic process using the example of its spread in other affected countries with similar socio-economic, natural-geographical, and other indicators [15, 16]. In this respect, it is particularly important to obtain information on the number and density of susceptible livestock in a certain administrative territory as well as the data on the location, number, and density of farms engaged in sheep and goat breeding, ownership forms, and the intensity of trade and economic relations with both the nearest neighbors and farms from other regions of the country.

**Figure 1:**
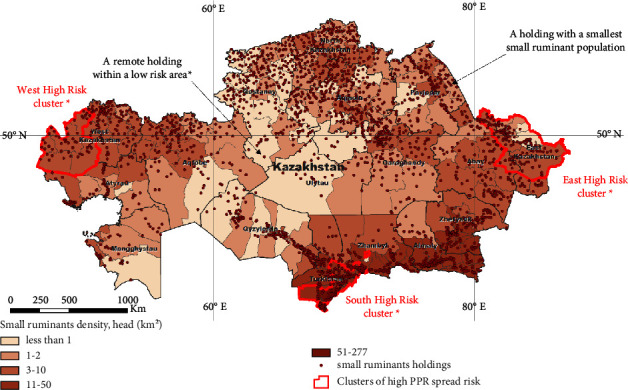
Small ruminant (SR) population density in the Republic of Kazakhstan, location of SR farms, and clusters of a high risk of the PPR spread as identified in the work of authors of reference [6]. The arrows indicate the farms used as the initial point of introduction of PPR virus in the different simulating scenarios.

In the study of [6], the regions within the country most susceptible to the spread of PPR in the case of disease introduction were identified (Figure 1). However, the results do not enable estimation of the scale of the potential disease spread within and beyond the risk regions, as well as the possible number of affected animals and the duration of the epidemic.

For the purpose of conducting a detailed analysis and consideration of possible scenarios of the spread of PPR in the territory of Kazakhstan, in this article, we undertook simulation modelling, which allows preliminary assessment of the degree of the impact of various factors on the likely level of spread of the disease in different areas of the country. Accordingly, the obtained data will increase the effectiveness of preventive measures and improve the preparedness levels to potentially prevent the introduction of the PPR virus into the territory of the country. This will serve as a basis for the National Veterinary Service to improve and implement veterinary surveillance measures, monitor the epizootic situation, and analyze the risk of the emergence and the disease spread.

## 2. Material and Methods

### 2.1. Study Area

**Figure 2:**
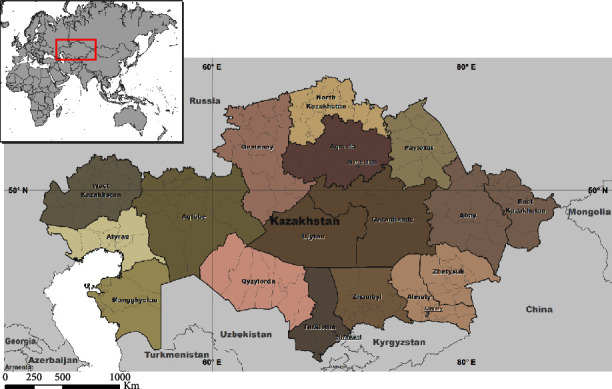
Geographical location and administrative division of the Republic of Kazakhstan.

The Republic of Kazakhstan (RK) is a state in central Asia and ranks 9th in the world in terms of territory and 63rd in terms of population. The territory of the country is divided into 20 first-level units (regions and federal cities) and into 224 second-level units (districts) (Figure 2).

### 2.2. Small Ruminant Population Data

Over a 3-year period (2018–2020), a nation-wide survey was undertaken in Kazakhstan in order to collect data on the location of livestock farms [6, 17]. This resulted in development of a database of small ruminant (SR) farms, including geographical coordinates and the population number in the farms (Figure 1). This database was used to simulate the spread of PPR between farms. In total, the database contains data on 2,478 SR farms of different ownership forms. The population size in the farms ranged from 18 to 167,918 animals. The median value was 4,213 heads. At present, the RK is free of PPR, so there are no approved veterinary and sanitary regulations for the prevention and the elimination of PPR. In particular, the ranking of farms according to biosecurity does not apply. Therefore, in this study, we considered all farms to be of the same type in terms of contact possibilities and biosafety level.

### 2.3. Simulation Method

The simulation approach based on a compartmental epidemic SIR model for individual farms was used in this article, taking into account the probability of disease spread between neighboring farms as well as indirectly between distant farms. This approach is based on the assumption that a farm (or “unit”) may be in one of the infection states at any given time: susceptible (S), latent (L), infected (I), or recovered (or destroyed-R). Depending on the condition at a given point in time, the probability of direct and indirect contact of a given herd with other herds in the region and consequently the probability of infection of these herds are assessed. The approach was implemented using the NAADSM (The North American Animal Disease Spread Model) simulation model [18–20]. The model also allows the inclusion of different control and prevention strategies (such as disease detection, contact tracing, zoning, and vaccination) and the assessment of their impact on the disease spread.

The application of the NAADSM model requires the specification of numerous disease parameters and the demographics of the susceptible population in the model region. Among the main parameters are the following: (1) the duration of the different stages of the disease (latent, subclinical, clinical, and immune); (2) the possibility of direct and indirect contact of the infected farm with other farms in the region; (3) distribution of distances between farms in the region. With the majority of parameters in the model being specified by means of distributions, it is possible to account for uncertainty in their numerical values and to carry out numerous iterations to obtain average parameters of the epizootic.

The model offers the possibility of simulating three ways of the disease spread between farms: (1) via direct contacts (movement or shipment of animals among units); (2) via indirect contacts (movement of people, materials, vehicles, equipment, animal products, etc., among units), as well as (3) by airborne spread. In our work, only the first two pathways were studied, as the airborne spread of PPR virus mainly occurs by droplets over short distances (about ten metres), i.e. predominantly within the herd [21–24].

### 2.4. Model Parametrization

Table 1 summarizes the values of the main parameters of the NAADSM model obtained majorly from a through literature review process and taking into account the specifics of herd management and veterinary legislation of the Republic of Kazakhstan. In addition, a number of other model parameters were estimated based on expert opinion and the experience of the authors.

In the simulation, we included “disease detection,” which represents detection and recognition of PPR clinical signs (with a probability increasing from 0 to 90% within 7 days of the unit being in clinical condition) and mortality from disease (with a probability increasing from 50% to 100% within 7 days after a unit is dead from disease).

Tracing of contact herds was not included in the model due to the lack of relevant regulations in the veterinary legislation of the Republic of Kazakhstan.

The modelling included the use of zoning after detection of the disease in the herd. The radius of the risk zone was assumed as 50 km, as recommended by the WOAH. Within the zone, restrictions on movement of animals and animal products are applied, with the effect of restrictions decreasing from 100% on day 1 after detection to 25% on day 7. In addition, it was assumed that the probability of detecting the disease in herds within the zone increases by 2 times (expert opinion).

According to the requirements of the veterinary legislation of the Republic of Kazakhstan, an infected herd has to be slaughtered after detection of the disease. An average depopulation rate of 10 herds per day was assumed.

After the infected herd has been identified, all other herds within a 50 km radius must be vaccinated. Delay in vaccination rollout was assumed to be one day. Vaccination capacity was assumed to be 100% of 10 herds per day.

In addition, the economic damage of the epidemic was assessed by considering three factors: (1) the cost of vaccination of one animal is 0.03 USD (at the exchange rate of the US Dollar to Tenge at the end of 2022); (2) the cost of slaughter of one animal is 4.6 USD; and (3) the cost of supervision and monitoring activities per animal is 7.2 USD. The cost of compensation of animal owners when flocks are culled and other costs related to detection and elimination of the outbreak were not taken into account.

### 2.5. Running the NAADSM Simulation

Simulation of the disease spread using the NAADSM model was performed using eight scenarios when one of the farms was assumed to be an index case.

Previously, three clusters were identified in the work of authors of reference [6] as being most susceptible to the disease spread in the Republic of Kazakhstan (Figure 1). We selected the smallest (in terms of livestock number) and the largest farms in each cluster as the initial index case. In addition, we considered as the initial index case the smallest farm in the Republic of Kazakhstan (18 head) and the most remote farm located in the area of the lowest exposure to the disease (according to the results of authors of reference [6]).

Simulation for each of scenarios was performed in 100 iterations, which allowed us to obtain the average values of epidemic indicators, as well as the boundaries of their 95% confidence interval. The introduction of the disease was simulated by assigning “latent” (L) status to the selected farm. Simulation continued until the end of the outbreak in each iteration (i.e., until the day when the number of latent farms becomes zero). The following epidemic indicators were recorded:

1. Total number of infected farms
2. Total number of animals in infected farms
3. Total number of farms where animals were slaughtered
4. Total number of animals slaughtered
5. Total number of vaccinated farms
6. Total number of animals in vaccinated farms
7. Total duration of the epidemic
8. The largest area within the designated risk zones
9. Total cost of animals slaughtering
10. Total cost of vaccination
11. Total cost of supervision in surveillance zones

### 2.6. Software

NAADSM v 4.0.13 software was used for simulation (https://www.naadsm.org).

The ArcMap Desktop 10.8.1 geographic information system was used to process and visualize geospatial data (ESRI, Redlands, CA, USA).

## 3. Results

Table 2 summarizes the results of the simulation of PPR outbreaks according to the eight scenarios.

Geographically, the epidemics did not extend beyond a local outbreak in any of the simulated scenarios, covering, at most, the territory of a neighboring district. The average area of the established risk zones does not exceed the average area of the administrative district, which is 35,568 km^2^, and the maximum area of the risk zones only exceeds this size in a few scenarios. The maximum number of infected farms in the most unfavorable outbreak scenario was 60, representing 2.4% of the total number of SR farms in the RK. The largest number of animals in the affected farms in the most unfavorable scenario was 5.5% of the total number of SR in all farms.

Table 3 summarizes the main economic indicators of epidemic: the cost of involuntary slaughter of animals and the cost of vaccination in the surveillance areas.

## 4. Discussion

The NAADSM model used for the simulation of the spread of PPR in the territory of the Republic of Kazakhstan in the case of introduction of the disease into different farms suggests that outbreaks are expected to be mostly of local scale without covering significant areas of the country, with adequate response measures—timely disease detection, zoning in accordance with the WOAH recommendations, and preventive vaccination of farms in the risk area.

Simulations did not reveal an obvious dependence of epidemic size and duration on the size of the farm being the index case of PPR. Only the dependence of the scale of the epidemic on the concentration and total number of small ruminants in the affected areas can be seen from our results. Thus, the largest number of affected farms is expected in the South risk cluster, i.e., the south areas of Turkistan and Zhambyl regions. The occurrence of PPR, even on the most remote farm in the lowest risk area, leads to a comparable number of affected farms as in the highest risk areas due to indirect contacts.

It is necessary to consider that the coverage of an area by PPR epizootic is significantly influenced by the distance distribution for indirect contact. Thus, the disease transmission with vehicles can occur at a distance significantly longer than 50 km, which can lead to the spread disease outside the surveillance area and significantly expand its geographical range. In this respect, the NAADSM model allows for rapid parameter changes and comparison of different scenarios on the principle of “what if....”

However, the following fundamental disadvantages and restrictions of both the applied model and its parameterization should be noted. Firstly, the model assumes isotropy over the entire simulating area, i.e. equal probability of the disease spread in any direction in any area. While in reality, the direction, in which the disease spreads, is mostly determined by transportation routes as well as by topographic features and natural barriers (rivers, mountains, forests, etc.). Secondly, the model contains a large number of initial parameters whose values can only be assessed approximately on the basis of expert opinion, the experience of the authors, or literature data, which may apply to conditions of another country. Thirdly, the assumption of equivalence of all farms in terms of biosecurity was used in our model, while in reality small farms may pose an increased risk of infection due to relaxed animal welfare regulations.

The estimated economic indicators derived in our simulating are purely indicative, as they do not take into account many components of economic damage such as payments to farmers and the organization of vaccination campaigns. Inclusion of such indicators is possible and requires the information on many additional costs that was not available during the preparation of this study.

Further work on simulating a potential PPR epidemic in Kazakhstan using this approach would mean, firstly, ranking all farms by number of livestock and appropriate safety measures defining the main indicators of the basic number of contacts. Consideration of farms' location in different climatic zones will also help in more adequate parametrization of farms in terms of specific livestock management practices that may influence their biosecurity. Secondly, more accurate parameterization of indicators of the impact of zoning on the number of contacts is needed. Thirdly, it is possible to include in the model those holdings that are not livestock farm but pose a risk in terms of the spread of PPR, such as abattoirs and livestock markets.

Taken as a whole, simulation results may be of interest to the Veterinary Service of the Republic of Kazakhstan in assessing the impact of different prevention and control strategies on the magnitude of a possible outbreak of PPR when the disease is introduced into the country.

## Figures and Tables

**Table 1:** Summary of NAADSM parameters on the PPR spread simulation in the Republic of Kazakhstan.

Parameter	Measurement unit	Value or distribution	Data sources
Duration of the latent period in PPR	Day	BetaPert (3, 7, 10)	[19, 25]
Duration of the subclinical period	Day	Not expressed	[19, 25]
Duration of the infectious clinical period	Day	BetaPert (2, 7, 21)	[26, 27]
Duration of the immune period	Day	BetaPert (180, 730, 1460)	[4]
Probability of the death of a herd from the disease	%	95%	[25]
Average number of contacts between herds by direct contact	Contacts per day	1	Estimates of experts
Probability of the disease transmission by direct contact	Probability	0.95	Estimates of experts
Distributing distances between herds for direct contact	Km	Lognormal (13, 10)	Calculated in GIS based on average distance between the nearest neighboring farms
Average number of contacts between herds by indirect contact	Contacts per day	0.5	Estimates of experts
Probability of the disease transmission by indirect contact	Probability	0.95	Estimates of experts
Distribution of distances between herds for indirect contact	km	Exponential (50)	Based on a recommended surveillance radius of 50 km, assuming that indirect contacts are most likely to occur within this radius
Duration of postvaccination immunity	Day	BetaPert (365, 720, 1460). Delay of 14 days after vaccination	[18, 28]
Probability of observing clinical signs after N-days in clinical condition	day-%	0–0%	Estimates of experts
		3.5–25%	
		7–90%	

**Table 2:** Summary output data of different scenarios of PPR spread in the Republic of Kazakhstan and epidemiological parameters (mean values and 95% CI boundaries).

Scenario	The initially infected farm	Total number of infected farms	Total number of infected animals	Total number of farms where animals were involuntarily slaughtered	Total number of animals involuntarily slaughtered	Total number of vaccinated farms	Total number of animals in vaccinated farms	Total duration of the epizooty, day	How many iterations out of 100 the zoning was established	The largest area of the risk zones (km^2^)
East high risk cluster	Small	5.29 (1–19.05)	11, 988 (34–48, 144)	3.58 (0–19.05)	9, 232 (0–48, 144)	4.64 (0–24.2)	12, 493 (0–74, 795)	33.6 ( 13–75)	39	16, 702 (0–29, 651)
	Large	4.2 (1–14)	43, 722 (24, 545–113, 773)	2.64 (0–14)	20, 888 (0–106, 056)	2.09 (0–14.05)	10, 648 (0–99, 817)	33 (14–73)	31	21, 028 (10, 462–31, 419)

South high risk cluster	Small	16.41 (1–51.05)	36, 8831 (565–1, 141, 021)	14.96 (0–49.15)	348, 226 (0–1, 135, 492)	15.8 (0–63.05)	387, 520 (0–1, 486, 300)	50.8 (14–109)	58	22, 626 (7, 725–41, 448)
	Large	14.53 (1–60)	418, 778 (167, 918–1, 240, 423)	13.2 (0–59.05)	317, 373 (0–1, 240, 423)	19.82 (0–67.05)	514, 865 (0–1, 713, 552)	47.2 (13–110)	41	28, 128 (7, 725–46, 777)

West high risk cluster	Small	6.31 (1–20)	37, 212 (2, 942–94, 563)	4.34 (0–19.05)	24, 905 (0–90, 557)	2.92 (0–19.05)	16, 746 (0–86, 787)	41.86 (14–96.1)	40	19, 425 (7, 339–33, 960)
	Large	3.59 (1–9)	90, 837 (43, 302–188, 813)	1.64 (0–8.05)	34, 981 (0–173, 407)	0.64 (0–3.15)	11, 012 (0–68, 376)	31 (12.95–61.05)	37	15, 223 (0–34, 989)

The smallest farm		4.67 (1–13.1)	10, 632 (18–27, 647)	2.79 (0–13.1)	6, 800 (0–25, 698)	3 (0–22.15)	9, 978 (0–61, 604)	55.6 (13.95–68.1)	29	22, 055 (9, 153–38, 422)
The most remote farm in the least risk area		4.76 (1–9)	23, 365 (4, 467–47, 495)	3.03 (0–9)	15, 758 (0–47, 336)	0.28 (0–1)	1, 065 (0–7, 784)	36 (12.95–65.05)	33	21, 292 (7, 725–36, 629)

**Table 3:** Summary output data of different scenarios of PPR spread in the Republic of Kazakhstan and economic parameters (mean values and 95% CI boundaries).

Scenario	The initially infected farm	Total cost of animals slaughtering (USD)	Total cost of vaccination (USD)
East high risk cluster	Small	26,616 (0–1,454,210)	2,572 (0–12,345)
	Large	28,483 (0–184,023)	2,034 (0–10,355)

South high risk cluster	Small	1,073,823 (0–3,325,365)	101,099 (0–331,022)
	Large	779,974 (0–35,526,118)	82,569 (0–344,176)

West high risk cluster	Small	44,258 (0–195,571)	3,249 (0–12,910)
	Large	69,943 (0–430,672)	3,496 (0–15,173)

The smallest farm		26,666 (0–199,110)	2,723 (0–19,263)
The most remote farm in the least risk area		41,407 (0–168,479)	1,245 (0–4,541)

## Data Availability

The data on the distribution of small ruminants' farms in the Republic of Kazakhstan are available from the corresponding author upon reasonable request. Public availability is not possible due to privacy reasons.
